# Downstream molecular pathways of FLT3 in the pathogenesis of acute myeloid leukemia: biology and therapeutic implications

**DOI:** 10.1186/1756-8722-4-13

**Published:** 2011-04-01

**Authors:** Shinichiro Takahashi

**Affiliations:** 1 The Division of Molecular Hematology, Kitasato University Graduate School of Medical Sciences, 1-15-1 Kitasato, Minami-ku, Sagamihara 252-0373, Japan; 2 The Division of Hematology, Kitasato University School of Allied Health Sciences, 1-15-1 Kitasato, Minami-ku, Sagamihara 252-0373, Japan

## Abstract

FLT3 is a type III receptor tyrosine kinase. Mutations of FLT3 comprise one of the most frequently identified types of genetic alterations in acute myeloid leukemia. One-third of acute myeloid leukemia patients have mutations of this gene, and the majority of these mutations involve an internal tandem duplication in the juxtamembrane region of FLT3, leading to constitutive activation of downstream signaling pathways and aberrant cell growth. This review summarizes the current understanding of the effects of the downstream molecular signaling pathways after FLT3 activation, with a particular focus on the effects on transcription factors. Moreover, this review describes novel FLT3-targeted therapies, as well as efficient combination therapies for FLT3-mutated leukemia cells.

## Introduction

FLT3 (Fms-like tyrosine kinase 3) is a member of the class III receptor tyrosine kinase family. Notably, approximately one-third of acute myeloid leukemia (AML) patients have mutations of this gene, and such mutations are one of the most frequently identified types of genetic alterations in AML. The majority of the mutations involve an internal tandem duplication (ITD) in the juxtamembrane (JM) domain of FLT3, which is specifically found in AML [1]. In accordance with the two-hit hypothesis [2] of leukemic transformation, FLT3-ITD expression in mouse bone marrow cells expressing a promyelocytic leukemia (PML)/retinoic acid receptor (RAR) α fusion protein of acute promyelocytic leukemia (APL) caused accelerated malignant transformation[3]. Indeed, FLT3-ITD is prevalent (~50%) in patients with translocations of t(15;17) [4]. In addition, frequent co-occurrence of mutations of FLT3 with mutations of nucleophosmin (NPM) [5] and DNA methyltransferase 3A [6] were reported in AML patients with normal karyotypes. These observations suggest that FLT3 mutations functionally cooperate with other molecules for leukemic transformation.

Based on these data and the literature, this review summarizes the current understanding of the prevalence, correlation with other molecular alterations, and intracellular downstream signaling pathways of FLT3 mutations. Moreover, the oncogenic effects of FLT3 mutations on myeloid transcription factors are also discussed. Furthermore, this review describes efficient combined molecularly-targeted therapeutic approaches for FLT3-activated AML cells.

### FLT3 structure and FLT3 ligand

**Figure 1:**
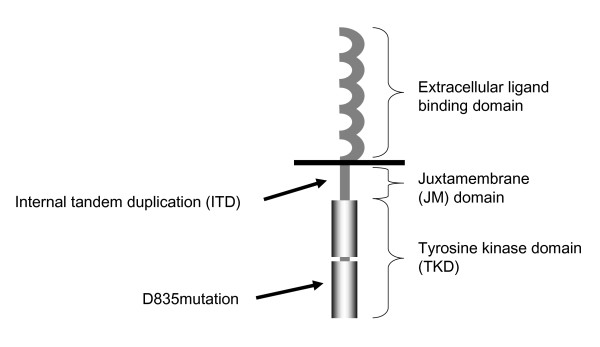
Schematic presentation of the FLT3 receptor.

The structure of FLT3 is shown in Figure 1. Two distinct classes of mutations have been identified in patients with AML, and the most common is an ITD in the JM region of the receptor [1]. Even though the ITD insertions vary in length, they always maintain a head-to-tail orientation and preserve the reading frame. It has been suggested that a conformational change in the JM domain is responsible for dimerization and receptor activation [7]. The second most common type of FLT3 mutations in AML are mutations in the activation loop of the tyrosine kinase domain (TKD) (Figure 1). Almost all of these mutations involve an aspartate-to-tyrosine substitution at codon 835, although other substitutions have also been identified [8,9]. These mutations cause a conformational change of the molecule and disrupt its autoinhibitory function, thereby rendering the receptor constitutively active [2,10,11].

The human *Flt3 *gene is located on chromosome 13q12 and encompasses 24 exons. It encodes a membrane-bound glycosylated protein of 993 amino acids with a molecular weight of 158-160 kDa, as well as a non-glycosylated isoform of 130-143 kDa that is not associated with the plasma membrane [10,12]. After the cloning of the *Flt3 *gene, soluble mouse Flt3 was used to clone the gene encoding the mouse Flt3 ligand (FL) [13]. The mouse FL cDNA was then used to clone the human *FL *gene [14]. The mouse and human *FL *genes encode proteins of 231 and 235 amino acids, respectively [15]. The cytoplasmic domains of murine and human FL show only 52% identity in the cytoplasmic domain. The *FL *gene encodes a type 1 transmembrane protein that contains an amino-terminal signaling peptide, four extracellular helical domains, spacer and tether regions, a transmembrane domain and a small cytoplasmic domain [15]. FL is expressed by most tissues, including hematopoietic organs (spleen, thymus, peripheral blood and bone marrow) and the prostate, ovary, kidney, lung, colon, small intestine, testis, heart and placenta, with the highest level of expression in peripheral blood mononuclear cells [11]. The brain is one of the few tissues without demonstrable expression of FL. Most immortalized hematopoietic cell lines express FL [11,16].

The expression of FL by a wide variety of tissues is in contrast to the limited expression pattern of FLT3, which is mainly found in early hematopoietic progenitor cells. These observations indicate that the expression of FLT3 is a rate-limiting step in determining the tissue-specificity of FLT3 signaling pathways.

### FLT3 mutations in hematopoietic malignancies

In 1996, Nakao *et al. *[17] found a unique mutation of FLT3 in AML cells. This mutation, comprising an ITD in the JM domain of the receptor (Figure 1), caused the coding sequence to be duplicated and inserted in a direct head-to-tail succession [17]. Subsequent studies showed that ITD mutations of the *FLT3 *gene occur in approximately 24% of adult AML patients [2]. In addition, activating point mutations of the FLT3 TKD, mainly at aspartic acid 835 (Figure 1), are found in approximately 7% of AML patients [9].

Since the first description, numerous studies have confirmed and extended these findings to the extent that FLT3 mutations are currently the most frequent single mutations identified in AML, and approximately one-third of AML patients have mutations of this gene [1,18]. FLT3-ITD mutations have also been detected in 3% of patients with myelodysplastic syndromes [1], and occasional patients with acute lymphoid leukemia [19,20] and chronic myeloid leukemia [21]. They have not been found in patients with chronic lymphoid leukemia, non-Hodgkin's lymphoma or multiple myeloma [1], or in normal individuals [22,23]. These findings suggest that FLT3 mutations have strong disease specificity for AML.

As a general rule, the presence of an ITD in adult patients seems to have little or no impact on the ability to achieve complete remission (CR). In children, however, several studies have reported a reduced CR rate [7,24]. The most significant impact of an ITD is its association with a higher leukocyte count, increased relapse risk (RR), decreased disease-free survival (DFS) and decreased overall survival (OS), which have been reported in most studies of children and adults aged less than 60 years [23]. Several groups found that an ITD is the most significant factor for predicting an adverse outcome in multivariate analyses [7,23,25,26]. In contrast, FLT3-TKD mutations tend to worsen the DFS and OS [9], although the differences are statistically significant for OS in patients aged less than 60 years [27]. In addition, it was reported that even in patients with normal cytogenetics and wild-type FLT3 (n = 113), clear tendencies for worse OS and event-free survival were found in patients with high FLT3 expression (n = 43) [28].

Falini *et al. *[5] described abnormal localization of NPM1 in AML patients. The C-terminus of this protein is mutated in approximately 27.5% of AML patients [29], and such mutations are probably the second most prevalent type of mutations in AML patients. A subsequent study suggested that NPM1 mutations are strongly associated with FLT3-ITD mutations in patients with a normal karyotype (NPM1-mutant/FLT3-ITD: 43.8% versus NPM1-wild-type/FLT3-ITD: 19.9%; P < 0.001) [29]. Quite recently, it was reported that Dnmt3A mutations were detected in 62 of 281 AML patients (22.1%), and these mutations were highly enriched in a group of patients with an intermediate-risk cytogenetic profile as well as FLT3 mutations (25 of 61 patients, 41.0%; P < 0.003) [6].

AML is a multistep process that requires the collaboration of at least two classes of mutations, comprising class I mutations that activate signal transduction pathways and confer a proliferation advantage on hematopoietic cells and class II mutations that affect transcription factors and primarily serve to impair hematopoietic differentiation [30,31] (Table 1). Hou *et al. *[32] investigated the prevalence and clinical relevance of mutations of PTPN11, which encodes human SHP2, and their associations with other genetic changes in 272 consecutive patients with primary AML. Among 14 patients with PTPN11 mutations, none had FLT3-ITD mutations. On the other hand, 6 of 14 patients with PTPN11 mutations had concurrent NPM1 mutations [32], suggesting PTPN11 is classified as a class I mutation molecule similar to the case for FLT3.

**Table 1:** A list of class I, class II and unclassified mutations

Class I mutations: Providing cellular proliferative and/or survival advantage	Class II mutations: Impairing cellular differentiation	Unclassified mutations:
Flt3 mutation	PML-RARα	NPM1
c-KIT mutation	AML1-ETO	Dnmt3a
N-or K-Ras mutation	CBFβ-MYH11	
PTPN11	AML1 mutation	
	C/EBPα mutation	
	MLL-PTD	

FLT3-ITD mutations are correlated with certain cytogenetic subgroups. Among APL patients with PML-RARα, it was reported that 30-50% of the patients had FLT3 mutations [4,27,33]. Frequent (~90%) co-occurrence was reported in patients with t(6; 9) and FLT3-ITD mutations [27,34]. Similarly, FLT3-ITD mutations are also frequently found in patients with mixed lineage leukemia (MLL)-partial tandem duplication (PTD) [35]. The rate of MLL-PTD in FLT3-ITD-positive patients was significantly higher than that in FLT3-ITD-negative patients [16/184 (8.7%) versus 32/772 (4.1%); P = 0.025] [35]. In analyses involving 353 adult *de novo *AML patients, Carnicer *et al. *[36] found cooperative mutations of FLT3-TKD with CBFβ/MYH11 rearrangement (four of 15 patients) and C/EBPα with FLT3-ITD (two of 82 patients). In comprehensive analyses of 144 newly diagnosed *de novo *AML patients, Ishikawa *et al. *[37] also found that most overlapping mutations consist of class I and class II mutations (Table 1). In addition to the frequent co-occurrence of FLT3 mutations with mutations of other molecules (e.g. NPM1, MLL-PTD, CBFβ/MYH11 rearrangement), they found that two of the 35 patients with FLT3 mutations also had AML1/ETO. Collectively, FLT3-ITD mutations play a key role in leukemogenesis by functionally cooperating with other molecules.

### Downstream pathways of normal FLT3

FL-mediated triggering of FLT3 induces receptor autophosphorylation at tyrosine residues, thereby creating docking sites for signal-transducing effector molecules and activating various signaling pathways. The downstream signaling cascade involves the tyrosine phosphorylation and activation of multiple cytoplasmic molecules. The FLT3 cytoplasmic domain physically associates with the p85 subunit of phosphoinositol-3-kinase (PI3K), Ras GTPase, phospholipase C-γ, Shc, growth factor receptor-bound protein (Grb2) and Src family tyrosine kinase, and results in the phosphorylation of these proteins [38]. These actions affect the activation of further downstream PI3K/protein kinase B (Akt) and mitogen-activated protein kinase (MAPK) pathways [39,40]. Bruserud *et al. *[41] reported that exogenous FL increases blast proliferation for not only patients with wild-type FLT3 but also patients with FLT3-ITD, as well as, FLT3-TKD mutations. Therefore, FL-mediated triggering of FLT3 appears to be important for both wild-type and mutant FLT3 signaling.

### Downstream pathways of oncogenic FLT3

FLT3-ITD mutations, as well as TKD mutations, result in the constitutive activation of FLT3 kinase. Mutations in the FLT3 JM domain and activation loop can be predicted to result in loss of the autoinhibitory function, with subsequent constitutive activation of FLT3 kinase and its downstream proliferative signaling pathways, including the Ras/MAPK kinase (MEK)/extracellular signal-regulated kinase (ERK) pathway and PI3K/Akt pathway [2]. In addition, and in contrast to wild-type FLT3 signaling, FLT3-ITD potently activates the STAT5 pathway [42-44]. STAT5 induces its target genes such as cyclin D1, c-myc and the anti-apoptotic gene p21, which are important for cell growth [45,46]. These effects may indicate a role of FLT3-ITD in the aberrant cell growth of leukemia cells [40,47]. In a microarray study using FLT3-ITD-expressing transgenic 32Dcl cells, the STAT5 target gene of a serine threonine kinase, Pim-2, was induced [43]. A different group reported that another serine threonine kinase, Pim-1, was upregulated by FLT3-ITD and is important for FLT3-ITD-mediated cell growth and anti-apoptotic effects [48]. Taken together, FLT3-ITD constitutively induces STAT5 and Pim serine threonine kinases, and their mechanisms may accelerate AML cell growth.

Sallmyr *et al. *[49] reported that FLT3-ITD mutations start a cycle of genomic instability whereby increased reactive oxygen species (ROS) production leads to increased DNA double-strand breaks (DSBs) and repair errors. They found that FLT3-ITD-transfected cell lines and FLT3-ITD-positive AML cell lines and primary cells exhibit increased ROS production. The increased ROS levels appear to be produced via STAT5 signaling and activation of RAC1, an essential component of ROS-producing NADPH oxidases. They provided a possible mechanism for the ROS generation because they found a direct association of RAC1-GTP binding to phosphorylated STAT5 (pSTAT5), and inhibition of the pSTAT5 level resulted in the decrease of ROS production. They concluded that the aggressiveness of the disease and the poor prognosis of AML patients with FLT3-ITD mutations could be the result of increased genomic instability driven by higher endogenous ROS, increased DNA damage and decreased end-joining fidelity. Further analyses from the same research group using FLT3-ITD-expressing cell lines and bone marrow mononuclear cells from FLT3-ITD knock-in mice demonstrated that the end-joining of DSBs occurs at microhomologous sequences, resulting in a high frequency of DNA deletions [50]. They found that the levels of Ku proteins, which are key components of the main nonhomologous end-joining (NHEJ) pathway, are decreased in FLT3-ITD cells. Concomitantly, the levels of DNA ligase IIIα, a component of alternative and less well-defined backup end-joining pathways, are increased in FLT3-ITD cells [50]. Cells treated with an FLT3 inhibitor exhibit decreased DNA ligase IIIα expression and a reduction in DNA deletions, suggesting that FLT3 signaling regulates the pathways by which DSBs are repaired [50]. Therefore, therapies to inhibit FLT-ITD signaling and/or DNA ligase IIIα expression may lead to repair that reduces repair errors and genomic instability.

It is notable that more than two-thirds of AML patients show FLT3 phosphorylation, even in the absence of activating mutations [51,52]. Increased FLT3 transcript levels are observed in a large number of AML samples, and this increased expression may also contribute to the phosphorylation of FLT3 and activation of its pathways [52]. Since several receptor tyrosine kinases are dimerized and activated even without ligand binding to their receptors [53], the upregulation of FLT3 may facilitate its dimerization and thereby enhance the phosphorylation. Meanwhile, Zeng *et al. *[51] demonstrated an increase in FLT3 autophosphorylation when leukemic blasts were incubated in medium for a while after being thawed, compared with washed newly thawed blast cells. Their findings indicate that the secreted soluble form of FL plays a role in cells with constitutive activation of wild-type FLT3.

### Inhibition of transcription factor functions by FLT3-ITD

Scheijen *et al. *[54] reported that FLT3-ITD expression in Ba/F3 cells resulted in activation of Akt and concomitant phosphorylation of the Forkhead family member FOXO3a. Phosphorylation of FOXO3a threonine 32 through FLT3-ITD signaling promotes their translocation from the nucleus to the cytoplasm. Specifically, FLT3-ITD expression prevented FOXO3a-mediated apoptosis and upregulation of *p27KIP1 *and *Bim *gene expression, suggesting that the oncogenic tyrosine kinase FLT3 can negatively regulate FOXO transcription factors through the phosphorylation of FOXO3a leading to suppression of its function, thereby promoting the survival and proliferation of AML cells [54].

FLT3-ITD is also known to inhibit the expression and function of several myeloid transcription factors. FLT3-ITD specifically inhibits the expression [55] as well as the function of C/EBPα through phosphorylation of the N-terminal serine 21 of this protein by activation of ERK [56]. Following this aberrant phosphorylation of C/EBPα, the differentiation of FLT3-ITD cells is blocked [56]. It was reported that mice carrying hypomorphic PU.1 alleles, which reduce PU.1 expression to 20% of the normal level, developed AML [57]. The expression of PU.1 is also significantly suppressed by FLT3-ITD [43,55]. In addition, the author's group previously reported that high expression of FLT3 is associated with low expression of PU.1 in primary AML cells [58]. These observations indicate that blockade of the function of myeloid transcription factors by FLT3 oncogenic signaling plays an important role in the pathogenesis of AML.

**Figure 2:**
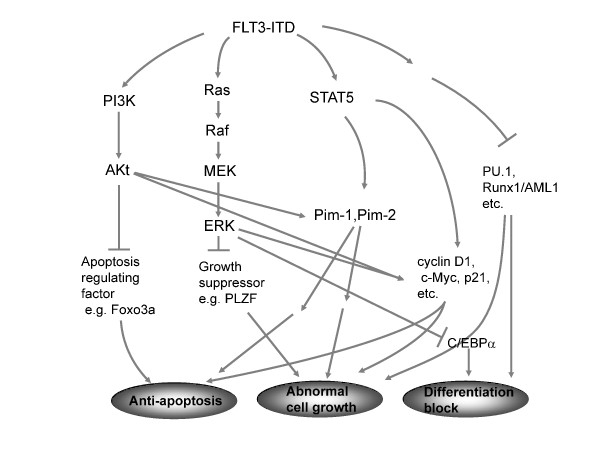
**Mechanisms of FLT3-ITD induced leukemogenesis**. Depicted is an outline of known pathways downstream of FLT3-ITD.

Silencing mediator of retinoic acid and thyroid hormone receptors (SMRT) recruits histone deacetylase (HDAC) and mediates transcriptional repression by interacting with various transcriptional repressors, including AML1-ETO [59], Runx1/AML1 [60] and promyelocytic leukemia zinc finger (PLZF) [47]. PLZF was identified as the translocation partner of RARα in t(11;17)(q23;q21) retinoid-resistant APL [61]. PLZF is expressed in myeloid progenitor cells and downregulated as the cells differentiate [61-63], suggesting an important role of PLZF in normal myeloid cell development. PLZF is a transcriptional repressor and a potent growth suppressor that blocks cell proliferation and myeloid differentiation through silencing of its target genes, including cell cycle regulators such as cyclin A2 [64,65]. The author and colleagues previously reported that FLT3-ITD expression dissociates PLZF and SMRT, and inhibits the function of PLZF, leading to aberrant gene regulation and abnormal cell growth in leukemia [47]. Runx1/AML1 is a Runt family transcription factor that is critical for normal hematopoiesis and regulates various genes as either a transcriptional activator or repressor [66]. Recently, it was reported that Runx1/AML1 functions as a senescence inducer [67]. Intriguingly, the author's group revealed that the Runx1/AML1-SMRT interaction is also disrupted by FLT3-ITD, leading to disruption of the function of Runx1/AML1 and aberrant expression of the Runx1/AML1 target gene p21^WAF1/CIP1 ^[60]. These findings are quite consistent with the notion of Yan *et al. *[68], who reported that disruption of the interaction between AML1-ETO and SMRT dramatically enhances the oncogenic potential of AML1-ETO. These findings are summarized in Table 2 and Figure 2. These observations indicate that inhibition of transcriptional repressor, growth repressor and senescence inducer functions through the dissociation of transcriptional repressors and co-repressors by aberrant FLT3-ITD signaling may another crucial mechanism for leukemogenesis.

**Table 2:** Inhibition of transcription factor functions by FLT3-ITD

Author	Target	Responsible signaling pathway	Mechanisms of the action
Mizuki *et al. *[43]	C/EBPα, PU.1	unknown	Down-regulates myeloid transcription factor C/EBPα and PU.1 expression.
Scheijen *et al. *[54]	FOXO3a	Akt	Inhibition of FOXO3a leads to the upregulation of *p27KIP1 *and *Bim *gene expression. These promote cell survival and proliferation.
Zheng *et al. *[55]	C/EBPα, PU.1	unknown	Down-regulates myeloid transcription factor C/EBPα and PU.1 expression. Those may play a role in myeloid differentiation block.
Radomska *et al. *[56]	C/EBPα	MEK/ERK	Phosphorylates serine 21 of C/EBPα, results in the differentiation block of MV4;11 cells.
Takahashi *et al. *[47]	PLZF	MEK/ERK	Dissociates its transcriptional co-repressor SMRT, inhibits the growth suppressor function of PLZF, leading to abnormal cell growth.
Takahashi *et al. *[60]	Runx1/AML1	unknown	Runx1/AML1-SMRT interaction is disrupted by FLT3-ITD, leading to the aberrant expression of the Runx1/AML1target gene p21^WAF1/CIP1^.

### FLT3-targeted therapies

The clinical outcome of AML was dramatically improved by the development of effective chemotherapy in the 1970s and subsequently improved by the development of hematopoietic stem cell transplantation therapy in the 1980s. However, the clinical outcome of AML has not improved since the 1990s, with the exception of the identification of all-trans-retinoic acid therapy for APL. Currently, highly specific molecularly-targeted therapies for AML cells are being investigated to further improve the clinical outcome of AML.

Since the identification of the high frequency of FLT3 mutations in AML, approximately 20 different experimental and/or clinical FLT3 inhibitors have been developed and described in the literature [69-82]. The compounds currently in development are heterocyclic compounds containing components that structurally mimic the purine ring of adenosine and become inserted into the ATP-binding site of FLT3 [69]. Among these compounds, SU11248 (sunitinib), MLN518 (tandutinib), CEP-701 (lestaurtinib) and PKC412 (midostaurin) have passed through preclinical studies and made the bench-to-bedside transition to clinical trials [69,82]. Although these inhibitors appear to have some activity as single agents, the responses to date have tended to be incomplete or of limited duration [83-86]. AC220 is a second-generation FLT3 inhibitor that appears to have excellent potency and selectivity for target inhibition *in vivo *[87]. Lestaurtinib trials have included extensive pharmacodynamic studies, and the data suggest that such first-generation FLT3 inhibitors inhibit their target in some but not all patients [82]. Although not definitive, such studies suggest the possibility that FLT3 inhibitors may have only a limited role as single-agent therapies, at least in patients with refractory or repeatedly relapsing AML. Although some patients with FLT3-ITD mutations can respond if adequate drug levels are achieved, a large number of patients are potentially resistant to the administration of single FLT3 inhibitors. These observations imply the presence of mechanisms by which leukemic blasts can evade the effects of FLT3 inhibitors [88]. The acquisition of secondary tyrosine kinase domain point mutations that interfere with drug binding is a well-documented phenomenon in CML patients receiving therapy with imatinib [89]. Preclinical studies using AML cell lines have shown that small variations in the molecular structure of the FLT3 activation loop can greatly influence the response to FLT3 inhibitors. Cells that express different FLT3-TKD mutations show distinctly different profiles of *in vitro *drug responses [90]. Cools *et al. *[91] described the results of an *in vitro *screen designed to discover mutations in the ATP-binding pocket of FLT3 that cause drug resistance, in which point mutations at four different positions were identified. These mutations conferred varying degrees of resistance to PKC412, with variable cross-reactivity observed for other inhibitors. Heidel *et al. *[92] reported the acquisition of a secondary FLT3-TKD mutation in a patient who responded to PKC412 but became resistant to the drug after 280 days of treatment. This patient was found to have developed a point mutation at one of the positions identified by Cools *et al. *[91], which had not been present at diagnosis. Research using FLT3 inhibitor-resistant leukemia cell lines generated through prolonged cocultures with FLT3 inhibitors has revealed that FLT3 inhibitor-resistant cells most frequently become FLT3 independent because of the activation of parallel signaling pathways that provide compensatory survival/proliferation signals when FLT3 is inhibited [93]. In resistant cells, FLT3 itself can still be inhibited but several signaling pathways normally switched off by FLT3 inhibition, including the PI3K/Akt and Ras/MEK/MAPK pathways, remain activated. Newly acquired activating NRAS mutations were found in two of the resistant cell lines, suggesting another means by which resistance may be acquired [93]. In addition, AML is a complex multigenetic disease and the simultaneous inhibition of other important tyrosine kinases, scaffolding proteins or relatively broad cytotoxic agents may be therapeutically advantageous as described in the next section.

### Development of efficient combination therapies for FLT3 mutated cells

In this context, several groups have recently reported that combinations of FLT3 inhibitor therapy and chemotherapy are synergistically effective [94-96]. Both CEP-701 and SU11248 have been investigated in combination with chemotherapy using *in vitro *models [94,95]. CEP-701 was found to induce cytotoxicity in a synergistic fashion with cytarabine, daunorubicin, mitoxantrone or etoposide when administered simultaneously with or immediately after the chemotherapeutic agent [94]. Additive or synergistic cytotoxic effects were also seen when model cell lines and primary blasts expressing FLT3-ITD mutants were simultaneously treated with SU11248 and daunorubicin or cytarabine [95].

The MEK/MAPK pathway is an important signaling cascade involved in the control of hematopoietic cell proliferation and differentiation [97,98]. Downregulation of MEK phosphorylation inhibits proliferation and induces apoptosis of primary AML blasts [99]. Consistent with these effects, the author found that inhibition of MEK/MAPK signal transduction strongly impairs the growth of FLT3-ITD cells [39]. Radomska *et al. *[56] recently reported the importance of inhibition of this pathway for not only cell growth but also restoration of the FLT3-ITD-mediated differentiation blockade of cells. These findings suggest that MEK is probably a good target for combination therapies with FLT3 inhibitors. Arsenic trioxide (ATO) has shown great promise in the treatment of patients with relapsing or refractory APL. It was recently reported that the combination of ATO with a MEK inhibitor is very efficient for not only APL blasts but also AML patients [100]. The author's group reported synergistic effects of ATO and MEK inhibition, as well as ATO and FLT3 inhibition, on FLT3-ITD cells [101]. The combination of ATO and AG1296, an FLT3 inhibitor, profoundly inhibited the growth and induced apoptosis of FLT3-ITD cells [101]. Common chemotherapeutic drugs usually have a wide range of cytotoxic effects on hematopoietic stem cells or progenitor cells of other tissues. In addition, there are many serious side effects of chemotherapy [102]. In contrast, the therapeutic dose of ATO used to treat APL is associated with an acceptable toxicity level without bone marrow hypoplasia or alopecia [103]. From these points of view, combination therapy with ATO may be advantageous for not only APL but also non-APL hematologic malignancies [104].

FLT3 has been shown to be a client protein for a chaperone, heat shock protein (Hsp) 90 [105]. Treatment with an Hsp90 inhibitor, such as herbimycin A, radicicol or 17-allylamino-demethoxy geldanamycin (17-AAG), was found to disrupt the chaperone association of FLT3 with Hsp90, thus directing FLT3 toward polyubiquitination and proteasomal degradation [106]. Hsp90 is likely to target misfolded proteins generated by mutations. It is therefore possible that FLT3-ITD proteins are unstable and require chaperoning by Hsp90 in leukemic cells. Consequently, combination therapy with an FLT3 inhibitor and an Hsp90 inhibitor, 17-AAG, was found to be effective against FLT3-ITD leukemia cells [107,108].

Chemokine stromal-derived factor 1a and its cognate receptor C-X-C chemokine receptor type 4 (CXCR4) were shown to act as critical mediators in stromal-leukemic cell interactions. CXCR4 is involved in the migration, homing and engraftment of AML cells to the bone marrow of NOD/SCID mice [109,110]. Intriguingly, CXCR4 expression was found to be significantly higher in FLT3-ITD AML samples than in FLT3-wild-type AML samples [111]. Targeting of CXCR4 may disrupt AML-niche interactions, sensitize leukemic blasts to chemotherapy and overcome cell adhesion-mediated drug resistance. Indeed, blockade of CXCR4 using small molecule inhibitors caused mobilization of resistant bone marrow leukemic blasts and was synergistic with conventional chemotherapeutics [112-114]. Therefore, targeting of CXCR4 in combination with FLT3 inhibitors may selectively eradicate FLT3-ITD cells. The development of these effective combination therapies against FLT3 activation may be the next breakthrough for AML therapy.

## Conclusions

Considerable progress has been made in our understanding of the molecular pathogenesis of AML, and numerous genetic abnormalities in AML have been identified. FLT3 is one of the key molecules with a role in the pathogenesis in AML. During the past decade, the function of the FLT3 pathway has been well characterized, and several FLT3 inhibitors have been developed. Nevertheless, the results of clinical trials of FLT3 inhibitors have only been partial and further precise studies for the FLT3 downstream pathways are required. Such analyses will hopefully lead to the development of effective therapies for AML in the future.

## Competing interests

The author declares that they have no competing interests.
